# State Aid Policies in Response to the COVID-19 Shock: Observations and Guiding Principles

**DOI:** 10.1007/s10272-020-0902-4

**Published:** 2020-07-28

**Authors:** Massimo Motta, Martin Peitz

**Affiliations:** 1 Barcelona Graduate School of Economics, Ramon Trias Fargas, 25–27, 08005 Barcelona, Spain; 2 Department of Economics, University of Mannheim, L 7, 3–5, 68161 Mannheim, Germany

## Abstract

As a general principle, state aid to firms and sector-specific support schemes should be used only when there are market failures; that is, when there are good reasons to believe that the market would not deliver efficient and/or equitable outcomes.

Thanks to COVID-19, markets have disappeared from one day to the next, and firms' assets in most sectors have been rapidly depleting. This has increased the need for many firms to obtain funding. However, the ongoing economic uncertainty has made it even more difficult for firms to obtain credit from the financial sector. Thus, firms that are profitable in normal times face liquidity problems as a result of a negative supply and/or demand shock, and the financial sector does not satisfy the individual needs for liquidity support because of the large macroeconomic risks. In such a case, governments have to step in and assist with liquidity support or the appropriate guarantees so that banks and other financial institutions can provide the needed liquidity. Governments may also design other support schemes that protect workers or help demand to recover. In the current crisis, there are no doubts that state support is necessary to avoid long-run consequences for firms, workers and their human capital.

Many countries, including most EU member states, have announced various measures (and are considering new ones) to control the public health crisis and address the economic fallout due to the COVID-19 pandemic. State aid can be seen as a response to a system failure resulting from a severe economic shock, either hitting one sector (with possible contagion effects in other sectors) or – as in the case of the coronavirus crisis – simultaneously hitting several sectors.

* This article is based on, but reorganises and adds to, Motta and Peitz (2020a,b).

**Massimo Motta**, ICREA – Universitat Pompeu Fabra, Barcelona; and Barcelona Graduate School of Economics, Spain.

**Martin Peitz**, University of Mannheim; and Mannheim Centre for Competition and Innovation, Germany.

As a general principle, state aid to firms and sector-specific support schemes should be used only when there are market failures; that is, when there are good reasons to believe that the market would not deliver efficient and/or equitable outcomes. Aid should also be effective and proportional to the aims it intends to achieve. While there seems to be wide agreement that government inaction is not an option during the COVID-19 crisis, a few observations may guide the design and revision of state support schemes.

## Sectors are hit differentially by the COVID-19 crisis

It has been documented that supply chain disruptions and demand shocks have had differential effects on sectors (for the UK, see for instance Bloom et al., 2020). This implies that some sectors need very little to no support, while others are in dire need. Clearly, liquidity support can then be targeted so that only those firms in need of such support sign up for the support programme. This implies that firms unaffected by the shock do not have the incentive or the ability to move under the umbrella of a liquidity support scheme. This also applies to state assistance for the labour costs of a firm (in particular, covering a fraction of the costs of furloughed employees). Keeping viable firms alive and enabling them to keep their staff makes it possible to quickly restart and scale up economic activities when demand picks up again and supply constraints have disappeared. By covering part of the wage bill for unemployed or underemployed staff, there is an incentive for firms hit by the shock to participate in this support scheme, while firms not hit prefer not to do so. Thus, well-designed liquidity support and employment subsidies can be applied across the whole economy, provided they are effectively targeted in the sense that only those firms negatively affected will participate in the programme.

## Some firms were struggling even before the COVID-19 shock

Some firms would have difficulties regardless, and the risk of a badly designed, overly generous support scheme is that it would keep those firms alive. The entry and exit of firms is an important process in any flourishing economy, as it leads to a better allocation of resources. Since such a view may be dismissed as 'neoliberal' in the public debate, it is important to reflect on what happens when non-viable firms are kept alive. Consider the following constructed example: a village has a zoning law in place such that two restaurants have a license to operate. Suppose that one of the restaurants serves lousy food and cannot pay its bills, while the other serves decent food. If the village authorities provide support to the former so that it can cover its losses, the villagers will continue to be served lousy food in this restaurant. If this restaurant were to exit the market, a different restaurant may serve the villagers better food. This increases the competitive pressure on the other restaurant and encourages it to strive even harder.

Therefore, state support schemes and in particular state aid that applies to a particular sector or to particular firms run the risk of supporting firms that are not viable in the long run even without the COVID-19 shock. It is therefore important that support schemes are temporary in nature. Also, to be eligible, well-established firms should provide evidence that their business was not in the red prior to the outbreak of the COVID-19 pandemic.

In line with these two observations, the European Commission (2020a, 2020b) adopted a Temporary Framework for state aid schemes aimed at ensuring firms' access to liquidity and finance, and at preserving employment. This framework provides some limiting principles, establishing the temporary nature of such public interventions, and favouring their effectiveness and their incentivising nature. For instance, firms that were already having difficulties on 31 December 2019, and hence *before* the crisis, cannot have access to most measures; credit guarantees for loans beyond €800,000 cannot apply to more than 90% of the loan; the loan principal should normally not go beyond certain amounts (25% of yearly turnover, or twice the yearly wage bill); and wage subsidies given to workers who would have otherwise been laid off because of the crisis should not exceed 80% of the monthly gross salary.

## Sectors and firms hit by a temporary shock may also be subject to a long-term shock

Some industries may never look the same after COVID-19. If large portions of temporary shocks become permanent, state aid will become more problematic for the sectors or firms that aim to preserve the status quo ante. Given the large fiscal strains on many countries, we submit that such support schemes for sectors that are unlikely to fully recover should not go ahead. We admit that such decisions are politically particularly hard to sell if the respective sectors are labour-intensive and have powerful trade unions or industry lobbies.

To the extent that this is foreseeable, support schemes should not use the status quo before the shock but rather the conditions that will prevail afterwards for reference. Thus, forward-looking state aid may also apply to sectors that were in decline before the shock or that will feel the long-run effects of the shock. Such sector-specific support schemes may include measures that facilitate scaling down and restructuring (e.g. a move away from fossil fuels in the case of the car industry). Such state aid has to be carefully designed so as to avoid spending funds on a lost cause and preserving an outdated industry structure.

## In the EU Single Market, some countries have more fiscal freedom for support programmes than others

In the EU context, there is the risk that public support for national companies creates trade and competition distortions within the internal market, and for this reason the European Commission (EC) has been given powers to control state aid. State aid programmes by EU member states require the approval of the EC. The founders of the EU understood very clearly that the internal market has to be protected from member states favouring their own companies, and introduced provisions in the Treaty on the Functioning of the European Union to this effect awarding the EC the task of state aid control.

The size of the economic shock and the ability to cushion its impact through state aid do not go hand in hand. In the current crisis, most countries hit severely by the COVID-19 pandemic are not in a strong fiscal position. This negatively affects the functioning of the Single Market. In particular, there is the risk of tilting the level playing field and creating a ‘domino effect’ (see Motta and Peitz, 2020a). If only some firms in a given industry are eligible for aid while others are not, competition will necessarily be distorted. This is inevitable when aid is provided by some countries and not by others, for instance, because only some member states can afford such aid or because different states support different industries. A firm that is generously funded by its home country becomes artificially more competitive, to the detriment of other efficient or more efficient rival companies, and the latter may be relegated to niche markets or even forced out of business. Or, to the extent that some of these rivals come from a home country that can afford state aid as well, a subsidy race among member states may be triggered, significantly wasting public money.

### Some viable businesses may need recapitalisation

The EC extended the state aid Temporary Framework well beyond liquidity support and employment preservation so as to include the recapitalisation of businesses (see European Commission, 2020c). In some circumstances, short-run liquidity support may not be enough and a lack of finance may have long-term consequences: a firm that just barely keeps up with its payment obligations may have to abandon or postpone investment and innovation plans. To the extent that such plans meet important EU policy objectives, for instance in energy transition and the digital agenda, aid that will enable their roll out may exceptionally be allowed (we proposed this in Motta and Peitz, 2020a; and this is also the position taken in European Commission, 2020c).

If recapitalisation takes the form of partial state ownership, as a matter of principle, this should be temporary and fully repaid shortly after the recovery of the sector, that is, after a period of a couple of years at most. Shares should be assessed at the market valuation *after* the crisis has hit but *before* the rumour of state aid support has spread. The longer the participation of the state, the bigger the dilution for current shareholders should be. (If a hybrid instrument allowing converting debt into equity is the chosen form of state support, similar principles should apply.) The EC has adopted these principles in the extension of the Temporary Framework (European Commission, 2020c).

Taking into account the arguments made above, a credible restructuring plan should be approved before any recapitalisation to ensure that public money does not support a level of activity by a firm or in an industry that is unlikely to be viable in the long run.

### A sector-specific demand-side stimulus has serious drawbacks

Another instrument to revive a sector is a demand-side stimulus, e.g. in the form of vouchers for particular purchases. Such an instrument has been used in the past to stimulate car sales and is also on the table in the aftermath of the COVID-19 crisis. There are several problems with a broad demand-side stimulus (e.g. covering car purchases broadly). First, demand expansion may be limited if vouchers are redeemed mostly by people who would buy anyway – e.g. if transaction prices are increased by the amount of the voucher, in which case the instrument simply leads to a cash transfer from the government to the firms in the sector. Second, if consumers pay less after redeeming the voucher and demand picks up, this increased demand may come at the loss of future demand because of intertemporal substitution. To a certain extent, such intertemporal substitution may be socially desirable, but it should be considered when introducing the subsidy.

Furthermore, a programme introduced in one member state but not in others may still be distortive even if applied to all purchases within the country in case there is a home bias in consumption. For example, the home bias is well documented in the car industry.

A voucher programme for an industry is an indirect subsidy to the firms in the industry. It may be popular as it could be presented as benefitting primarily consumers. Industry lobbyists also prefer it as the firms operating in the industry may get the support with few strings attached (e.g. on managerial compensation and dividend policies). Strings can more easily be attached when state aid goes directly to firms. It should be stressed that a voucher programme might have further pitfalls. For instance, if vouchers for the purchase of cars running on fossil fuels were introduced, this would also conflict with the EU's climate objectives and other environmental goals (e.g. the reduction of nitrogen oxide emissions).

## EU-wide sector support system would avoid the distortions created by national programmes

A truly European public support programme would not suffer from risks to the internal market's functioning as funding decisions would be made at the European level and based on common goals. Moreover, all companies operating in a sector covered by such a programme could be beneficiaries, independent of the country they originate from (Motta and Peitz, 2020a). In line with this observation, the European Commission (2020c) stated that

> [i]f support were to be granted at EU level, taking into account the EU common interest, the risk of distortion to the Internal Market could be lower, and may therefore require less stringent conditions to be imposed. The Commission considers that additional EU level support and funds are necessary to make sure that this global symmetric crisis does not transform into an asymmetric shock to the detriment of Member States with less possibility to support their economy and the EU's competitiveness as a whole. (C164/4)

In some countries, some individual companies are particularly close to political decision-making and may lobby for particularly generous support programmes with few strings attached. While an individual company's influence at the member state level may be strong, its position is much weaker at the EU level. This provides another strong argument in favour of an EU-wide programme, as the EC is less likely to be captured by special interests than individual member states. For the sake of well-functioning economies in all member states, it would help if they publicly acknowledged the advantages of EU-wide programmes.

An advantage of an EU-wide sector support system compared to national programmes is that all firms in that particular sector would be eligible for aid, which would eliminate a source of distortion, namely, that only firms from some member states (and possibly the wrong ones) may receive aid within the sector.

One of the advantages of the EC playing a central role in designing a European aid programme is that it would reduce horse-trading between member states. The track record of the EC in this regards gives some reasons for hope: the EC has (in general) been able to resist the recurrent pressure for it to relax state aid control over the years.

In addition to competition policy objectives, there are other policy objectives that are linked to EU-wide goals and may justify a leadership role by the EC. Individual member states may not have the resources or, because of cross-country externalities, may not be willing to provide sufficient resources to pursue other objectives, such as climate and digital ones, or may lack resilience in times of crisis that would generate benefits in other member states as well (see Bénassy-Quéré et al., 2020; and Motta and Peitz, 2020b). State aid in the member states and EU funding schemes should also be aligned with those goals.
